# Green Total Factor Productivity Growth: Policy-Guided or Market-Driven?

**DOI:** 10.3390/ijerph191710471

**Published:** 2022-08-23

**Authors:** Shuai Wang, Cunyi Yang, Zhenghui Li

**Affiliations:** 1 School of Econimics, Central South University of Forestry and Technology, Changsha 410004, China; 2 Lingnan College, Sun Yat-sen University, Guangzhou 510275, China; 3 Guangzhou Institute of International Finance, Guangzhou University, Guangzhou 510405, China

**Keywords:** green total factor productivity, environmental regulation, marketization, PSTR

## Abstract

The green growth mode of modern economy is affected by both policy and market, but previous studies have lacked a comparison between the two effects on green economy development. Which is the leading factor of green growth: policy or market? Using the Panel Smooth Transition Regression (PSTR) model and the twelve-year data of more than 200 prefecture-level cities in China, we compared and analyzed the linear and non-linear effects of environmental regulation and marketization degree on green total factor productivity (GTFP). The results show that: (1) both environmental regulation and marketization degree have a non-linear promoting effect on GTFP. (2) GTFP is mainly market-driven rather than policy-guided. (3) Environmental regulation and marketization promote the improvement of GTFP through the industrial upgrading effect and the innovation development effect, respectively. This paper makes up for the comparative analysis gap of factors in the field of green growth and extends from the single determination of influencing factors to the importance of the comparison of influencing factors with the transition perspective. The conclusions provide a reference for the green development of countries and regions, emphasizing the importance of green development policies adapting to local conditions and time and providing evidence for market-oriented green economy development.

## 1. Introduction

Green development has gradually become a global consensus on sustainable economic development. It emphasizes the dynamic balance of economy, society and environment within a region, thus ensuring intergenerational equity. All countries in the world have attached great importance to the green development strategy. The 193 member states of the United Nations formally adopted the “Transforming Our World: 2030 Agenda for Sustainable Development” at the World Summit on Sustainable Development [1]. However, according to the conclusion of the “Global Environment Outlook”, the realization of environmental sustainability is not satisfactory, and problems such as the continuous deterioration of the ecological environment, the unsustainable use of natural resources, and climate change are still prominent [2]. After the reform and opening-up, China’s economy has undergone rapid development for more than 30 years, and the average annual GDP growth rate has exceeded 10%. However, while China’s economy continues high-speed growth, it also pays too much attention to speed and ignores the quality of economic development. The extensive economic development mode of “high input, high consumption, high emission and low efficiency” has caused a waste of resources and severe damage to the environment and ecology. Judging from the experience of developed countries in pollution control, China has to change its extensive economic development mode of sacrificing the environment for rapid development. The report of the 19th National Congress of the Communist Party of China proposed to focus on high-quality economic development. Therefore, the determination of how to coordinate the relationship between economy and ecology and improve the quality of economic growth is the primary task facing China’s development at the current stage.

In this context, it is of great practical significance to clarify the relationship between green total factor productivity (GTFP) and environmental policies and markets, which is conducive to the high-quality development of the world economy and China’s economy under the “New Normal”. The essence of improving the quality of economic growth is to improve energy efficiency and reduce environmental pollution in economic development. Compared with total factor productivity (TFP), green total factor productivity (GTFP) incorporates energy consumption and environmental pollution into the analysis framework of economic growth [3,4]. It is more reasonable to use it to evaluate economic quality under the constraints of resources and environment. In order to control environmental pollution, local governments have promulgated and implemented various environmental regulations, reducing environmental pollution to a certain extent. However, there are also some environmental governance measures at the expense of developing or transferring high-polluting industries, deviating from the original intention of improving the quality of economic growth. From the perspective of reducing costs and avoiding policy and moral hazards, enterprises in the market will also spontaneously improve green technology to achieve more environmentally friendly production. Therefore, in terms of increasing GTFP, it is worth exploring and comparing the influence effects of environmental regulations and the market. We can put forth the following questions: how do policies and markets affect GTFP? Are they linear? What is the relation between the two impacts? By what mechanisms do policies and markets affect GTFP?

Research on GTFP is deepening with the increasing environmental problems [5,6,7,8,9]. For the measurement of GTFP, the traditional TFP growth index ignores undesired outputs, which may lead to biased evaluation. Pittman first applied data envelopment analysis and considered undesired outputs [10]. In order to reveal the influence of undesired outputs on TFP, Chung et al. and Fare et al. proposed the directional distance function and constructed the Malmquist Luenberger index, which is more in line with the concept of environment [11,12]. However, since then, most studies have been limited to radial and directional methods, which cannot effectively overcome measurement errors caused by these methods. Therefore, Fukuyama and Weber established a generalized SBM directional distance function based on non-radial and non-directional methods [13]. Later, Oh constructed a global production possibility set and proposed a GML index that can be used to evaluate green total factor productivity [14]. However, both the SBM directional distance function and the GML index have certain defects when they are used separately. Therefore, many scholars have used the GML index based on the SBM directional distance function to measure GTFP in recent years. As for the influencing factors of GTFP, some scholars analyzed the impact of economic development on GTFP, such as Zhang et al., who verified the inverted U-shaped relationship between economic development and GTFP [15].

Policy guidance is an important factor affecting GTFP. At present, there are mainly the following three views: The first is that environmental regulations will increase the cost of environmental compliance for companies, reduce energy efficiency and corporate performance, and reduce GTFP, a phenomenon known as the “cost effect” of environmental regulations [16]. The second view holds that reasonable environmental regulations will promote innovation by offsetting the increased costs of regulations, thereby promoting technological progress, improving energy efficiency, and increasing GTFP, namely the “compensation effect” of environmental regulations [17,18,19]. The third view believes that the cost effect and the compensation effect coexist, and the impact of environmental regulations on GTFP depends on which effect dominates under different intensities of environmental regulations. On the one hand, short-term “cost effects” crowd out innovation investment and reduce corporate productivity. In the long run, rational manufacturers will use the “compensation effect” of technological innovation to offset the cost increase caused by the “cost effect” and improve corporate productivity, such as improving production and pollution control technology. Therefore, environmental regulations with a certain intensity can stimulate technological innovation and improve GTFP [20]. Environmental regulations have a “U”-shaped non-linear relationship with GTFP. On the other hand, high-intensity environmental regulations can affect production activities. There is an inverted “U”-shaped changing trend between environmental regulations and GTFP, so environmental regulations should be within an appropriate range; too high or too low a regulatory level is not conducive to the improvement of GTFP [21].

Academic circles have already begun to pay attention to the theory of environmental policy evaluation. In terms of the theory of evaluation criteria, multiple criteria are adopted. The evaluation standard refers to the scale according to which the policy is evaluated and is the basis of evaluation work [22]. There is no absolute authoritative evaluation standard for environmental policy evaluation, but as one of the public policies, we can refer to the evaluation standard of the classical theory of public policy evaluation [23]. According to the public policy evaluation theory, scholars generally evaluate from the perspective of effectiveness, efficiency, and fairness. The environmental performance index (EPI) has been established internationally to measure the environmental performance level of countries [24]. In the theory of the evaluation method, the cost–benefit analysis method is adopted. Environmental policy assessment methods include the social investigation assessment method [25], the environmental economic assessment method [24], the prediction analysis method, the comparative analysis method [26], the statistical analysis method, the analytic hierarchy process, and the comprehensive assessment method [27]. Environmental Kuznets curves show that when the economic development reaches a certain level, that is, after reaching a certain critical point or “inflection point”, with the further increase in per capita income, the environmental pollution turns from high to low, the degree of environmental pollution gradually slows down, and the environmental quality gradually improves [28].

To sum up, the previous empirical literature mainly focused on the impact of environmental regulations on GTFP while ignoring the important driving force of the market on the improvement of GTFP, leaving room for research. Therefore, starting from how to optimize the balance between economic development and environmental pressure, this paper provides a new perspective for the impetus of GTFP, that is, to explore whether GTFP is policy-guided or market-driven, as well as the mechanism.

The possible marginal contributions of this paper are as follows: First, it examines the impact of policy guidance and market orientation on the GTFP of prefecture-level cities. Unlike other literature that separately studied the relationship between GTFP and various influencing factors, this paper compares the effects of policy and market factors on GTFP. Second, it reveals the impact mechanism of environmental policy on GTFP. Existing research generally believes that environmental regulations can squeeze out innovation costs in a short time, while ignoring the impact of industrial changes caused by policy changes. This paper expands related research and improves the explanatory power of existing theories. Third, this paper adopts the PSTR model for research. Most of the previous studies have mainly used methods such as the DID. This paper can better study the non-linear changes in policy and market.

**Figure 1:**
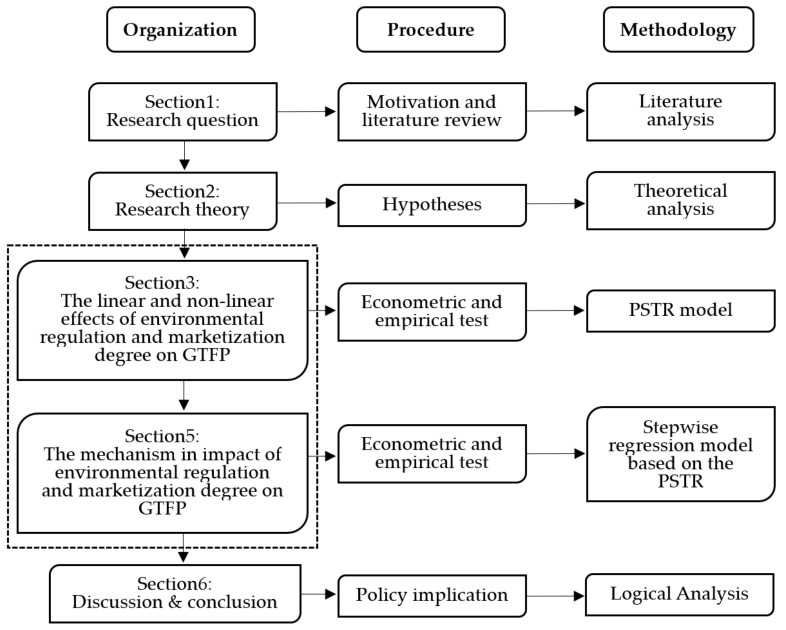
Methodological flowchart.

The rest of this paper is arranged as follows: Section 2 designs a model and introduces data; Section 3 tests the linear and non-linear effects of environmental regulation and marketization degree on GTFP; Section 4 further explores the two impact mechanisms; and Section 5 summarizes the conclusions. Figure 1 is the methodological flowchart.

## 2. Research Design

### 2.1. Hypothesis

First of all, the relationship between environmental regulations (ERs) and GTFP has been debated in academia. Due to the complexity of the issue itself, it is difficult to give a simple answer, and conclusions vary from different perspectives. On the one hand, environmental regulations have a technology-spillover effect, which can promote the transformation of industrial structure through technology empowerment, reduce the proportion of manufacturing, and increase the proportion of the service industry, thereby realizing the improvement of green total factor productivity. From this perspective, there is a linear relationship between ERs and GTFP [29,30]. On the other hand, the environmental Kuznets curve theory points out that there is an inverted U-shaped relationship between economic development and environmental pollution. When a country’s economic development level is low, environmental pollution will gradually deteriorate with economic development and per capita income increase. When economic development reaches a certain level, or a certain critical point or “inflection point,” environmental pollution will gradually slow down, and environmental quality will improve with economic development and the increase in per capita income. This phenomenon is known as the environmental Kuznets curve [28]. The theory of the environmental Kuznets curve inspires us to think about whether there is a similar relationship between environment, market, and GTFP. Based on this, the following hypothesis is proposed in this paper:

**H1.**  *Both environmental regulations and marketization have non-linear effects on GTFP.*

Secondly, marketization can promote economic growth, while the role of environmental policy on economic growth is controversial. At present, the research on the impact of marketization on GTFP is very limited, but from the perspective of the impact on economic growth, research found that marketization can improve resource allocation and accelerate economic growth [31]. The impact of economic systems and policies is extensive [32,33,34]. It is generally believed that environmental policy is the main factor leading to the increase in enterprise costs and negative impacts on productivity and competitiveness [20,35]. The Porter Hypothesis holds that appropriate environmental regulations can encourage enterprises to carry out more innovative activities, which will improve the productivity of enterprises, offset the costs caused by environmental protection, and improve product quality and profitability in the market, which may enable domestic enterprises to obtain competitive advantages in the international market [36]. Therefore, if the policy effect is stronger than the market effect, the critical point is that environmental policy can stimulate enterprise innovation and further improve production efficiency [37]. Based on this, this paper puts forward the following hypothesis:

**H2.**  *The impact of marketization is greater than that of environmental regulation.*

Innovation and industrial upgrading are the two major driving forces for the development of the green economy [38]. According to China’s National Bureau of Statistics, on the one hand, China’s economic development is primarily driven by the improvement of industrial upgrading momentum: both high-tech manufacturing and high-tech service industries have maintained rapid growth, and investment in high-tech industries is also accelerating. The driving effect of industrial upgrading will still support the development of China’s economy [39,40,41]. On the other hand, innovation-driven development also plays an increasing role in supporting the economy. Some green, low-carbon, and smart products are growing well [42]. The output of products such as integrated circuits, new energy vehicles, and solar cells has maintained rapid growth, which will also help support economic development.

This paper mainly analyzes the mechanism of environmental regulations affecting GTFP from two aspects. On the one hand, environmental policies have led to the closure of polluting enterprises, thereby optimizing the industrial structure. In theory, the proportion of polluting enterprises in the broad industrial sector can be gradually reduced through industrial structure optimization, because industrial structure optimization means that the proportion of the tertiary industry represented by technology-intensive industries increases, and such industries are relatively clean industries with high efficiency and low emissions. Their substitution for polluting enterprises is conducive to the growth of GTFP. Based on this, this paper puts forward the following hypothesis:

**H3.**  *Environmental regulations improve GTFP through the effect of industrial upgrading*.

This paper analyzes the mechanism of marketization degree affecting GTFP from two aspects. On the one hand, marketization is bound to bring about competition. After considering additional non-linear factors, some scholars have found a significant inverted U-shaped relationship between competition and innovation; that is, higher competition initially increases innovation speed and then reduces it [43]. On the other hand, the empirical conclusions of innovation’s effect on GTFP are complicated, but in general, innovation plays a leading role in promoting the growth of GTFP in China [44]. Based on this, the following hypothesis is proposed:

**H4.**  *Marketization improves GTFP through the innovation development effect*.

### 2.2. Model

According to the existing research, it can be inferred that the impact of environmental policies and market factors on green development may not be a single linear relation. Taking environmental policy as an example, environmental policy can promote green economic development. However, when the level of environmental policy is too low or the intensity of environmental policy is too high, the problem of “ignoring policy” or “tyrenny is filler than tiger” will appear in the process of green development, which will inhibit the development of the real green economy, that is, there is an optimal “threshold” in theory. Therefore, this paper adopts the PSTR model proposed by González et al. and Fouquau et al. to compare the non-linear effects of policy and market factors on urban GTFP [45,46]. The advantages of this model are as follows: (1) Compared with the ordinary linear panel model, the PSTR model can better describe the relationship between economic variables by identifying the heterogeneity of the cross-section data according to the non-linear statistical test, thus reflecting the slow change of the system through the transition variables. (2) The PSTR model can be regarded as a generalized panel threshold regression (PTR) model. Although the PTR model can also reflect cross-sectional heterogeneity, it is only suitable for economic data with structural mutations. The PSTR model has a broader scope of application. It is suitable for the continuous and smooth economic data of regime transitions. Therefore, it is more reasonable to use the PSTR model to study the non-linear effects of policy and market factors on urban GTFP.

The basic PSTR model with a single transition formula is defined as follows:

(1) $$y_{it}=\mu_{i}+\beta_{0}^{\prime }x_{it}+\beta_{1}^{\prime }x_{it}G\left(q_{it};\gamma ,c\right)+\varepsilon_{it}$$

where i represents different individuals; t represents different time periods; $y_{it}$ is the dependent variable; $x_{it}$ is the k-dimensional time-varying exogenous explanatory variable; $\mu_{i}$ and $\varepsilon_{it}$ represent the individual fixed effect and the random disturbance term, respectively.

The main feature of the PSTR model is the transition function $G\left(q_{it};\gamma_{j},c_{j}\right)$. It is a bounded and continuous function of the transition variable $q_{it}$, and its value is normalized between 0 and 1, allowing the model’s coefficient estimates to transit smoothly between $\beta_{0}\sim \left(\beta_{0}+\beta_{1}\right)$. The transition function usually takes the form of a logical function. According to the definition of the transition function by Gonzalez et al., the following formula is obtained:

(2) $$G\left(q_{it};\gamma_{j},c_{j}\right)=\frac{1}{\left[1+exp\left(-\gamma_{j}\prod_{j=1}^{m}\left(q_{it}-c_{j}\right)\right)\right]}$$

where $c={\left(c_{1},\cdots ,c_{m}\right)}^{\prime }$ is an m-dimensional vector of location parameters, and parameter γ determines the slope of the transition function. In practice, m=1 or m=2, since these two cases contain common types of variation in parameters. When m=1 and γ→∞, the PSTR model changes to a two-regime PTR model, because when γ→∞, the transition function $G\left(q_{it};\gamma_{j},c_{j}\right)$ changes to a characteristic function; when $q_{it}>c_{j}$, the value of the transition function is 1; otherwise, it is 0. When m=2, the transition function takes a minimum value at the point $\left(c_{1}+c_{2}\right)/2$, and when the value of the transition variable $q_{it}$ is low or high, the transition function’s value is 1. At this time, if γ→∞, the model becomes a three-regime PTR model whose outer regimes are identical and different from the mid-regime. Generally, when m>1 and γ→∞, the number of identical regimes remain two, but the function switches between 0 and 1 at $c_{1},\cdots ,c_{m}$. Additionally, when γ→0, the transition function $G\left(q_{it};\gamma_{j},c_{j}\right)$ is constant and Formula (1) degenerates into a homogeneous or standard linear model with fixed effects.

The PSTR model of the non-linear impact of policy and market factors on urban GTFP constructed in this paper is as follows:

(3) $$GTFP_{it}=u_{i}+\beta_{1}\;ER_{it}+\sum_{k}\beta_{k}X_{it}+\left(\beta_{1}^{\left(1\right)}\;ER_{it}+\sum_{k}\beta_{k}^{\left(1\right)}X_{it}\right)*G\left(q_{it}^{\left(1\right)};\gamma^{\left(1\right)},c^{\left(1\right)}\right)+\varepsilon_{it}$$

(4) $$GTFP_{it}=u_{i}^{\prime }+\beta_{1}^{\prime }\;MAR_{it}+\sum_{k}\beta_{k}^{\prime }X_{it}+\left(\beta_{1}^{{\left(1\right)}^{\prime }}\;MAR_{it}+\sum_{k}\beta_{k}^{{\left(1\right)}^{\prime }}X_{it}\right)*G\left(q_{it}^{{\left(1\right)}^{\prime }};\gamma^{{\left(1\right)}^{\prime }},c^{{\left(1\right)}^{\prime }}\right)+\varepsilon_{it}^{\prime }$$

In Formulas (3) and (4), i refers to the individual city, and t represents the time year; GTFP is the explained variable green total factor productivity; ER and MAR are the core explanatory variables, representing the government environmental regulation index and the degree of marketization, respectively; $X_{it}$ is a series of control variables that affect urban GTFP; $u_{i}$ represents the individual fixed effect; and $\varepsilon_{it}$ represents the random disturbance term.

### 2.3. Variables

Based on data availability, this paper selects data from 284 prefecture-level cities in China as research objects, including 3408 samples from 2008 to 2019. The explained variable is green total factor productivity [47,48,49], and the core explanatory variables are the government’s environmental concern and the degree of marketization. Data envelopment analysis (DEA) is a method of operational research and studying economic production boundary. This method is generally used to measure the production efficiency of some decision-making departments [50,51,52,53]. The green total factor productivity (GTFP) data are calculated by the DEA model based on the super-efficiency SBM-GML [54,55,56,57,58]. The input variables are urban fixed-asset investment, employment, built-up area, and energy consumption; the expected output is GDP. The undesired outputs are waste water, waste gas, and solid emissions. This paper refers to the research of Chen et al. to measure the government environmental regulation stringency (*ER*) 27. First, keywords related to environmental protection are extracted from multiple environmental policy reports and speeches. Second, the annual government work reports of 284 prefecture-level cities across the country during the survey period are collected. Finally, the text analysis is used to measure the proportion of sentences related to environmental protection; then, by multiplying this by 100, the urban environmental regulation stringency of Chinese prefecture-level governments (*ER*) is obtained. The marketization degree (*MAR*) is measured by the relative proportion of employment in private and individual sectors to the total population. 

The control variables are: the level of regional urbanization (*urb*), which is calculated by the ratio of the population of the municipal district to the total population of the city [59]; the degree of openness (*ope*), which is represented by the ratio of the city’s actual foreign capital to GDP; population density (*den*), which is measured by the logarithm of the population per square kilometer of the city; the traffic development level (*tra*), which is calculated by dividing the total passenger volume of the city by the total population, and then taking the logarithm; the postal development level (*pos*), which is calculated by dividing the urban postal service income by the total population and then taking the logarithm. 

The mediating variables are: industrial structure upgrading (*ind*), calculated by the proportion of the added value of secondary and tertiary industries in GDP; the regional innovation and entrepreneurship index (*inn*), referring to the National Development Institute of Peking University. The index (*inn*) includes five indicators of the number of new enterprises, attracting foreign investment, attracting venture capital, the number of patent authorizations, and the number of trademark registrations. The data of the control variables are from the “Urban Statistical Yearbook of China”, and some missing values are interpolated according to the changing trend. All data are normalized to the same dimension (0 to 1) using a normalization method. Table 1 reports descriptive statistics and data sources for all variables in this paper.

## 3. Results

### 3.1. The PSTR Test

Before using the PSTR model to study the relationship between GTFP and the government environmental regulation index as well as the marketization degree, the model needs to be tested for heterogeneity to determine whether there is a non-linear relationship between the two. If not, a linear panel regression model should be used.

First, the linear test of the PSTR model (1) is carried out, and the null hypothesis of the test is that since there are uncertain parameters in the PSTR model, it cannot be tested. The solution of Gonzalez et al. is used to perform a first-order Taylor expansion of the transition function $G\left(q_{it};\gamma ,c\right)$ in the model at γ=0 to further obtain the auxiliary regression:

(5) $$y_{it}=\mu_{t}+{{\beta_{0}}^{\prime }}^{*}x_{it}+{\beta_{1}^{\prime }}^{*}x_{it}q_{it}+\cdots +{\beta_{m}^{\prime }}^{*}x_{it}q_{it}^{m}+\varepsilon_{it}^{*}$$

The parameter vector $\beta_{1}^{*},\cdots ,\beta_{m}^{*}$ is the multiplication term of γ. $=+R_{m}\beta_{1}^{\prime }x_{it}$. $R_{m}$ is the residual of the Taylor expansion. Therefore, testing the null hypothesis $H_{0}:\gamma =0$ in Model (1) is equivalent to testing $H_{0}^{*}:\beta_{1}^{\prime *}=\cdots =\beta \prime_{m}^{*}=0$ in Model (5). Based on this, we set $SSR_{0}$ as the panel residual sum of squares when the null hypothesis $H_{0}^{*}$ holds and $SSR_{1}$ as the panel residual sum of squares under the alternative hypothesis. Then, we construct the LM statistic, its F form, and the LRT statistic to test the null hypothesis $H_{0}^{*}$.

(6) $$LM=\frac{TN\left(SSR_{0}-SSR_{1}\right)}{SSR_{0}}\sim \chi^{2}\left(mk\right)$$

(7) $$LM_{F}=\frac{\left(SSR_{0}-SSR_{1}\right)/mk}{SSR_{0}/\left(TN-N-mk\right)}\sim F\left(mk,TN-N-mk\right)$$

(8) $$LRT=-2\left[\log\left(SSR_{1}\right)-\log\left(SSR_{0}\right)\right]\sim \chi^{2\left(mk\right)}$$

where *TN* represents the total sample of the regression; k represents the number of explanatory variables; *LM* and *LRT* obey the Chi-square distribution; and $LM_{F}$ obeys the F distribution. Next, the number of transition functions and location parameters in the PSTR model should be determined to determine the parameters *r* and *m*. When m = 1 or m = 2, the parameter r is determined by performing the non-linear test on the remaining parameters. The specific method is similar to the homogeneity test. Table 2 and Table 3 show the results of the linear test of the model and the non-linear test of the remaining parameters, respectively.

From the linear test results in Table 2, it can be seen that for the two models with independent variables as ER and MAR that no matter whether m=1 or m=2, the test results of LM, $LM_{F}$, and LRT can reject the null hypothesis $H_{0}:r=0$ at the 1% significance level, indicating that the PSTR model has at least one threshold, which means that there is heterogeneity in the panel data in this paper. Therefore, it is necessary and reasonable to study the non-linear relationship between environmental regulation, marketization degree, and GTFP with the help of the PSTR model.

The remaining non-linearity test in Table 3 can further determine the number of transition functions. In the case of m = 1, the model with independent variables ER and MAR cannot reject this null hypothesis $H_{0}:r=1$ at the significance level of 10%, indicating that the number of transition functions is 1. Similarly, in the case of m = 2, r = 2 is selected for the model, whose independent variable is *ER*, and r = 1 is selected for the model, whose independent variable is MAR.

Parameter m is determined according to AIC and BIC information criteria and whether location parameter c is within the search range. According to Table 4, for the model with the independent variable *ER*, the parameters r = 2, m = 2; for the model with MAR as the independent variable, the parameters r = 1, m = 2.

The estimation results of the PSTR models with independent variables ER and MAR and the fixed-effect model are shown in Table 5 and Table 6, respectively. β represents the parameter estimation results of the linear part of the model. $\beta^{\left(1\right)}$ and $\beta^{\left(2\right)}$ stand for the parameter estimation results of the non-linear part of the model. According to the setting rules for the search range of location parameters in this paper, the range of ER location parameters is [0.027, 0.795], and the range of MAR location parameters is [0.004, 1], both of which are within the range.

**Figure 2:**
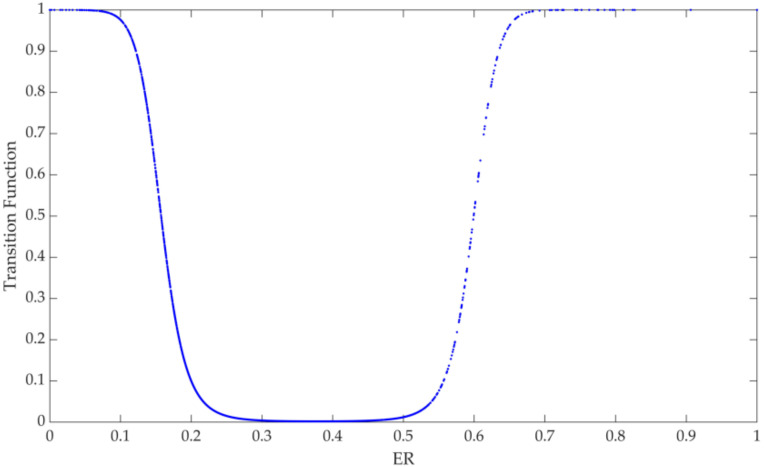
Transition function figure of ER.

The image of the transition function $G_{1}=(1+$ of the PSTR model of ER shows a trend of first decreasing, then becoming stable, and then increasing (see Figure 2). When the environmental regulation level is equal to 0.371 ($i.e.,\;(c_{1}+c_{2})/2$), the minimum value is close to 0, and the non-linear impact of the model is the least. It can be seen from Table 5 that the linear and the non-linear estimation coefficients of ER are significantly positive, indicating that for any level of government environmental regulation, ER has a significant promoting effect on GTFP. In addition, γ=129.2577 shows that the speed of the model transition between different systems is very fast, and the transition function shows a rapid change trend. The PSTR model of ER is a three-regime model with identical outer regimes on both sides and a different mid-regime. There are differences in the promotion degree of ER to GTFP. When ER is less than 0.325 and greater than 0.416, the promotion effect of ER on GTFP is more obvious under the outer regime, and when ER is between 0.325 and 0.416, the transition function value is close to 0, and its promoting effect on GTFP is mainly reflected in the linear part. The promoting effect of ER of the mid-regime on GTFP is weaker than that of the outer regimes on both sides.

The regression results of the fixed-effect model show that the coefficient of ER is significantly positive, which further verifies that government environmental regulations have a significant promoting effect on green total factor productivity. From the control variables, *den* and *pos* are significant in both the linear and non-linear parts of the model (1), indicating that no matter what the level of ER is, population density has an inhibitory effect on green total factor productivity ($\beta +G_{1}\left(ER;\gamma ,c_{1},c_{2}\right)<0$), and the postal development level has a promoting effect on green total factor productivity ($\beta +G_{1}\left(ER;\gamma ,c_{1},c_{2}\right)>0$). *urb* is only significantly negative in the linear part, indicating that the inhibitory effect of the urbanization level on GTFP is not affected by ER. *tra* is only significantly negative in the non-linear part, indicating that the level of traffic development only has an inhibitory effect on GTFP when the level of ER is extremely low or high.

**Figure 3:**
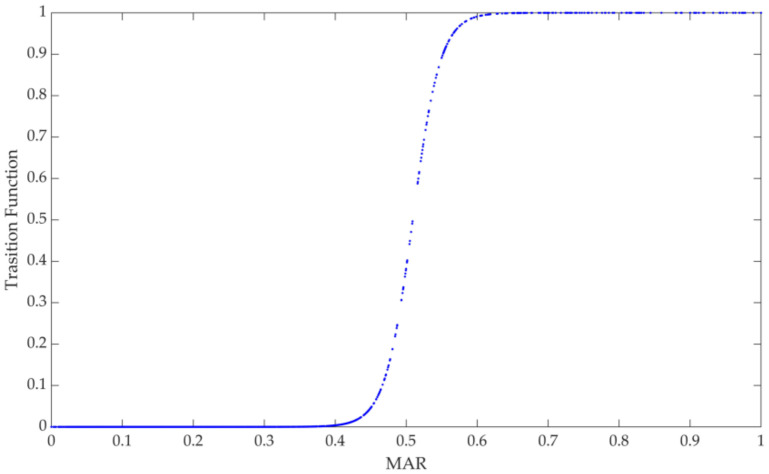
Transition function figure of MAR.

For the PSTR model of MAR, the image of the transition function $G_{2}\left(MAR;\gamma ,c_{1}\right)={\left(1+\exp\left(-\gamma \left(MAR_{it}-c_{1}\right)\right)\right)}^{-1}$ with one location parameter presents an “S” shape (see Figure 3). When the value of MAR is at a low level, the value of the transition function is close to 0, and its effect on GTFP is mainly reflected in the linear part. It can be seen from Table 5 that both the linear and non-linear estimation coefficients of MAR are significant, indicating that for any degree of marketization, MAR has a significant promoting effect on GTFP. There are differences in the promotion of GTFP by different degrees of MAR. The threshold for changing the speed of MAR improvement is 0.5092; that is to say, when MAR is greater than 0.5092, its speed of promoting GTFP improvement is significantly increased. In addition, the slope parameter γ=47.7281, and it can also be seen from Figure 3 that the transition speed of the model between different regimes is fast, and the transition function presents a rapid changing trend.

The regression results of the fixed-effect model show that the coefficient of MAR is significant, which further verifies that the degree of marketization has a significant promoting effect on green total factor productivity. As for the control variables, the linear and non-linear parts of *ope* and *den* are both significant, indicating that the promotion effect of openness on GTFP gradually weakens with the increase in MAR, and population density has an inhibitory effect on GTFP when the MAR level is low. With the increase in the MAR level, the inhibitory effect of population density on GTFP turns into a promotion effect and continues to increase. *tra* is only significantly positive in the linear part, indicating that no matter what the level of MAR is, the level of traffic development has a promoting effect on GTFP. *pos* is only significantly positive in the non-linear part, indicating that the postal development level has no significant effect on GTFP when the MAR level is low, but with the improvement of marketization, its role in promoting GTFP continues to strengthen.

### 3.2. Results Analysis

Government environmental regulations are often mandatory. They intervene in the environment by formulating relevant laws, regulations, and environmental standards to control the “three wastes” emissions of enterprises, and less consideration is given to the emission reduction capabilities of enterprises and regions in the implementation process. In the period when the government environmental regulation is weak, often, in the early stage of policy implementation, in order to attract more enterprises and regions to promote GTFP jointly, the government will adopt certain incentive measures. At this time, the base increases, and the emission of “three wastes” begins to decrease, having a very obvious effect on the improvement of green total factor productivity. In the period of strong government environmental regulation, the government has higher requirements for standards and stricter punishments, and the marginal cost of traditional end-of-pipe governance gradually increases. Enterprises need to increase the R&D of cleaner production and energy-saving and emission-reduction technologies to reduce costs. In this process, heavily polluting enterprises and extensive industries will face the pressure of transformation or even elimination, ultimately promoting the overall green total factor productivity. In the transitional stage of these two periods, although government environmental regulations have a certain role in promoting the efficiency of GTFP, the overall enthusiasm of enterprises and regions and the strictness of policy implementation are relatively weak. Therefore, in the transitional period, government regulations will reduce the promotion of GTFP. In addition, it can be seen from Figure 2 that most of the samples are in the outer regimes, indicating that China’s environmental regulations can effectively promote the improvement of green total factor productivity, and the environmental regulation system has gradually entered the stage of strategic transformation, and various environmental protection regulations have gradually formed. In addition, the coverage of environmental regulations and the degree of compliance of enterprises is relatively high, and China’s economic development is heading towards the coordinated development of economy and environment.

The degree of marketization reflects the enthusiasm of enterprises to actively improve green total factor productivity. Enterprises regard the environment as a type of production factor, and changes in factor prices can be transmitted to green total factor productivity through enterprise costs and benefits. The green tax rate represented by the environmental protection tax can be regarded as the external cost brought by environmental factors. To maximize profits, enterprises choose to innovate energy-conservation and emission-reduction technologies at the production end or control pollution at the end of production to achieve green total factor productivity improvements. In addition, rewards related to environmental protection can also encourage enterprises to innovate, produce an “innovation compensation effect”, and effectively stimulate the motivation of enterprises to improve green total factor productivity. The enthusiasm of enterprises to take the initiative to improve green total factor productivity is driven by the continuous reduction in tax costs and environmental incentives and compensation, resulting in a large-scale competitive effect in the market. After the marketization reaches a certain threshold, the promotion effect on GTFP will be further improved. From Figure 3, it can be seen that the distribution of samples in the low and high regimes is similar, indicating that enterprises have gradually participated in the GTFP improvement team independently and regard environmental protection as their future development planning and social responsibility. To sum up, we accept Hypothesis 1.

Combining the empirical results of the linear and the non-linear parts, the effect of market driving on green total factor productivity is greater than that of government policy guidance. Therefore, we accept Hypothesis 2.

## 4. Additional Analysis

Further analysis of the impact mechanism of environmental regulations and marketization degree on GTFP will help to clarify the source of the difference in the non-linear impact and driving force of the two. Based on the theoretical hypothesis analysis above, government environmental regulations improve green total factor productivity through the industrial upgrading effect, and marketization improves green total factor productivity through the innovation development effect. Therefore, we refer to the practice of Zhong et al. to construct a stepwise regression model based on the PSTR [60]:

(9) $$GTFP_{it}=u_{i}+\beta_{1}\;ER_{it}+\sum_{k}\beta_{k}X_{it}+\left(\beta_{1}^{\left(1\right)}\;ER_{it}+\sum_{k}\beta_{k}^{\left(1\right)}X_{it}\right)*G\left(q_{it}^{\left(1\right)};\gamma^{\left(1\right)},c^{\left(1\right)}\right)+\varepsilon_{it}$$

(10) $$\;M_{it}=u_{i}^{\prime }+\beta_{1}^{\prime }\;ER_{it}+\sum_{k}\beta_{k}^{\prime }X_{it}+\varepsilon_{it}^{\prime }$$

(11) $$GTFP_{it}=u_{i}^{\prime \prime }+\beta_{1}^{\prime \prime }\;ER_{it}+\beta_{2}^{\prime \prime }\;M_{it}+\sum_{k}\beta_{k}^{\prime \prime }X_{it}+\left(\beta_{1}^{{\left(1\right)}^{\prime \prime }}\;ER_{it}+\beta_{2}^{{\left(1\right)}^{\prime \prime }}\;M_{it}+\sum_{k}\beta_{k}^{{\left(1\right)}^{\prime \prime }}X_{it}\right)*G\left(q_{it}^{{\left(1\right)}^{\prime \prime }};\gamma^{{\left(1\right)}^{\prime \prime }},c^{{\left(1\right)}^{\prime \prime }}\right)+\varepsilon_{it}^{\prime \prime }\#$$

In Formulas (9)–(11), $M_{it}$ represents the mediating variables, including the industrial upgrading indicator ind and the regional innovation and entrepreneurship indicator inn. When testing the effect of market-oriented innovation and development, we replace $ER_{it}$ with $MAR_{it}$. The meanings of other symbols are consistent with those in Formulas (1)–(4). Here, we focus on the significance and values of the regression coefficients $\beta_{1}^{\prime \prime }$ and $\beta_{1}^{{\left(1\right)}^{\prime \prime }}$ in Formula (10): if they are significantly positive and numerically less than $\beta_{1}$ and $\beta_{1}^{\left(1\right)}$, there is a certain degree of mediating effect; if they are not significant, but $\beta_{2}^{\prime \prime }$ and $\beta_{2}^{{\left(1\right)}^{\prime \prime }}$ are significant, this indicates that the mediating variable has played a full mediating role [61,62].

According to the steps of Formulas (9)–(11), we test the industrial-upgrading effect in policy guidance on GTFP, and the results are shown in Table 7.

Government environmental regulations improve GTFP through the industrial upgrading effect, simultaneously reflected in both the linear and non-linear parts. From the regression coefficients in Table 7, it can be seen that after adding industrial upgrading as a mediating variable, the regression coefficient of ER is not significant, and the regression coefficient of the industrial upgrading is significantly positive. The industrial upgrading shows a full mediating effect in ER affecting GTFP; that is, the improvement of government environmental regulations improves green total factor productivity mainly by promoting industrial upgrading. This may be because government environmental regulations are mandatory and have the “one size fits all” feature. In the long run, the traditional governance cost of enterprises will exceed their affordable range, so they seek to increase the R&D of cleaner production and energy-saving and emission-reduction technologies. In this process, some enterprises that fail to achieve industrial upgrading in time will face elimination, ultimately promoting the improvement of green total factor productivity in the market. Therefore, Hypothesis 3 is accepted.

Similarly, according to the steps of Formulas (9)–(11), we test the effect of innovation development in the market driving GTFP, and the results are shown in Table 8.

Marketization improves GTFP through the innovation development effect, and it is mainly reflected in the linear part. As Table 8 shows, after adding the mediating variable: the regional innovation and entrepreneurship index, the regression coefficient of MAR in the linear part is significantly positive, but it is reduced compared with that before adding the mediating variable, indicating that the innovation development performs a partial mediating effect in market driving GTFP. The improvement of marketization can increase the occurrence of innovation activities in the region. By increasing R&D funds and recruiting innovation talents, enterprises can achieve technological innovation to reduce emission-reduction costs and improve green total factor productivity. Driven by the market, enterprises continue to iterate and update emission-reduction technologies. Through the demonstration effect and the spillover effect of innovation, innovation activities in the region can be comprehensively improved, thereby improving green total factor productivity. Therefore, Hypothesis 4 is accepted.

By comparing the industrial upgrading effect with the innovation development effect, it is not difficult to find why the market-driven promoting effect on GTFP is dominant. The green growth of modern economy is a growth mode based on technological innovation and characterized by industrial structure transformation and upgrading. Under market conditions, the economic transformation of less developed regions is accompanied by the mismatch between human capital and industrial structure, so their industrial policies should prioritize industrial upgrading rather than innovation driving to lead to appropriate matching between human capital and industrial structure through industrial upgrading. At the beginning of the reform and opening up, under the guidance of an export-oriented economic development strategy, China made full use of the cost advantage due to its rich labor resource endowment and successfully promoted the transition from the industrial structure dominated by heavy industry in the era of the planned economy to the market-oriented one dominated by light industry. With remarkable achievements in the industrial structure upgrade and services accounting for a surge year by year, the overall industrial structure is already relatively high, and the effect of promoting green development by upgrading the industrial structure is weak. In contrast, China’s innovation and development capacity are still at a level that does not match its economy, and vigorously developing innovation is the top priority. Therefore, we believe that from the perspective of green development, the market-driven innovation development effect is a more crucial sustainable development path.

## 5. Discussion and Conclusions

Which is the primary driver of green total factor productivity between environmental regulation and marketization? Based on the data of 284 prefecture-level cities in China for 12 years, this paper employed the PSTR model to compare and analyze the impact of government environmental regulation and marketization on GTFP and the mechanisms by combining the linear and non-linear perspectives. We obtained the following main conclusions through theoretical analysis and empirical tests.

First, both government environmental regulations and marketization have a non-linear promotion effect on GTFP. The test results show that the impact of government environmental regulations and marketization on GTFP has both a linear part and a non-linear part. Among them, the linear part is significantly positive. The non-linear impact of government environmental regulations on GTFP shows a trend of first decreasing, then stabilizing, and then increasing. The non-linear impact is significantly lower than the linear impact. The non-linear effect of marketization on GTFP presents an “S” shape. When the value of MAR is at a low level, the value of the transition function is close to 0, and after crossing the threshold, the speed of marketization promoting GTFP increases significantly. The non-linear impact is also significantly lower than the linear impact. In long-term green development, policy and market are the two main driving sources. Policy subjectively guides the economy to transform towards long-term sustainable development, while the market follows its objective laws to gradually eliminate inefficient industries and guide the real economy towards high-quality development. However, their roles in promoting green development will change with the change in the times and objects [63,64,65,66].

Second, green total factor productivity is mainly affected by marketization rather than environmental regulations. By comprehensively analyzing the linear and non-linear parts of the impact, we found that no matter at which location the transition function is, the comprehensive impact capacity of marketization (linear + non-linear) is greater than that of environmental regulation, mainly because the linear impact intensity of marketization is large. In the long run, marketization is bound to improve resource allocation and production efficiency, so as to speed up the economic development toward the sustainable goal. However, due to the limitations of subjective inference and time lag, it is difficult to effectively achieve the original intention of the policy-makers through environmental regulations, and they may even be mismatched with the actual situation. At present, with the slow development of the world economy, most regions and countries are not sensitive to environmental policies. The market factor of objective development is the fundamental driving force of green development [67,68].

Finally, environmental regulation and marketization improve green total factor productivity through the industrial upgrading effect and the innovation development effect, respectively. Industrial upgrading plays a full mediating role in policy guiding green development, while innovation development plays a partial mediating role in market driving green development. The mediating effect research provides a way to understand the relationship between the two. In the process of rapid industrialization and modernization, China’s industrial structure upgrading has achieved remarkable results, the proportion of the service industry has increased year by year, and the overall industrial structure has been high. The path of industrial structure upgrading is no longer sensitive to the effect of green development. In contrast, improving overall production efficiency through innovation development is an enduring issue of green development [69].

The conclusions of this paper provide references for national and regional green development. Firstly, the green effect of the current policy guidance is weak, presenting a non-linear relationship in which the outer regimes have a significant impact, and the middle regime has a small one; therefore, governments can consider adjusting the existing green development policy system to reduce the impact of insufficient policy sensitivity. In order for green development policies to play a better role in adjusting economic structure, governments can consider changing the existing long-term green development plans into short-term ones, strengthening and improving precise and targeted supporting policies. If the policy system can be divided into more levels, and supporting policies such as targeted incentives, performance appraisal preference, and profit loss compensation can be further improved, the incentive effect on green development can be further generated. 

Secondly, regions should actively guide the private economy to reform the backward situation of marketization, strengthening the transmission efficiency of market-driven green development. The low level of marketization in some regions weakens the transmission efficiency of green development. The government can promote the marketization transformation of underdeveloped regions by guiding private capital spillover from developed regions. On the one hand, some developed regions have made great progress in marketization transformation, and private capital from these high-quality markets will greatly assist the marketization transformation of less developed regions. On the other hand, due to the small market size, it is difficult for the less-developed regions to bring into play the scale-economy scale effect of marketization transformation. Private capital in high-quality markets will simultaneously enhance the scale effect and development space of both regions. 

Thirdly, regional green development should pay close attention to the two main transmission channels of industrial upgrading and innovation development. Green development is a necessary requirement for building a high-quality modern economic system and a fundamental solution to the pollution problem. The focus will be on adjusting the economic structure and making innovations in energy technologies, fostering and strengthening energy conservation and environmental protection industries, and realizing the cyclic connection between the production and the living systems. Nowadays, the ecological environment has become an essential part of national and regional comprehensive competitiveness. Protecting the environment is to protect productivity. By optimizing the industrial structure and taking the development road of science and technology leading, resource-saving, and ecological protection, we can realize the transformation from the “dilemma” between economic development and environmental protection to the “win-win” of the coordinated development of the two, thereby achieving the synchronous promotion of economic construction and ecological construction.

Admittedly, this paper also has some shortcomings. (1) Although this paper uses a large number of urban samples and many original indicators for testing, it is always based on historical data. The empirical results can reflect the relation between past variables, but they do not mean that the future situation can be fully predicted. (2) The PSTR model cannot solve the endogenous problem and can only rely on panel models to alleviate some of the endogenous problems. However, this statement is generally not convincing nowadays. The solution of endogenous problems still needs to find tool variables or other methods. (3) Because the test in this paper is based on the dimension of many years at the city level, many data related to green growth cannot be fully included in the control variables of this paper.

In future research, firstly, scholars can consider studying the prediction effect of environmental policy and market driving on green development from the perspective of time series model or the machine learning method. Secondly, the PSTR model cannot solve the endogenous problem, requiring scholars to go further in variable processing and eliminate the influence of endogenous variables from environmental policy and market-driven variables. Finally, scholars should be encouraged to go to the grass-roots level for investigation, collect first-hand and original data, and enrich the depth and width of research in the field of green development.

## Figures and Tables

**Table 1:** Variable descriptive statistics and data sources.

Variable	Observation	Mean	Standard Deviation	Q25	Q75	Source
GTFP	3408	0.260	0.172	0.145	0.310	Web search through Python
ER	3408	0.301	0.127	0.211	0.374	DEA calculation
MAR	3408	0.210	0.190	0.089	0.252	Urban Statistical Yearbook of China
urb	3408	0.330	0.248	0.143	0.442	Urban Statistical Yearbook of China
ope	3408	0.089	0.092	0.019	0.128	Urban Statistical Yearbook of China
den	3408	0.654	0.145	0.571	0.768	Urban Statistical Yearbook of China
tra	3408	0.417	0.122	0.346	0.478	Urban Statistical Yearbook of China
pos	3408	0.309	0.142	0.216	0.375	Urban Statistical Yearbook of China
ind	3408	0.746	0.161	0.649	0.871	Urban Statistical Yearbook of China
inn	3408	0.517	0.286	0.269	0.768	National School of Development

**Table 2:** Linearity test results.

Independent Variable	$H_{0}:r\;=\;0,\;H_{1}:r\;=\;1$					
	m = 1			m = 2		
	LM	$LM_{F}$	LRT	LM	$LM_{F}$	LRT
ER	24.229 *** (0.000)	3.721 *** (0.001)	24.315 *** (0.000)	56.423 *** (0.000)	4.366 *** (0.000)	56.895 *** (0.000)
MAR	20.313 *** (0.001)	3.740 *** (0.002)	20.374 *** (0.001)	69.961 *** (0.000)	6.527 *** (0.000)	70.689 *** (0.000)

Notes: *p* values are in parentheses, *** indicates significance at the 1% level.

**Table 3:** Remaining non-linearity test results.

Independent Variable	*m* = 1					
	$H_{0}:r\;=\;1,\;H_{1}:r\;=\;2$			$H_{0}:r\;=\;2,\;H_{1}:r\;=\;3$		
	LM	$LM_{F}$	LRT	LM	$LM_{F}$	LRT
*ER*	3.513 (0.742)	0.534 (0.783)	3.515 (0.742)	-	-	-
*MAR*	7.737 (0.171)	1.415 (0.216)	7.746 (0.171)	-	-	-
**Independent Variable**	m = 2					
	$H_{0}:r\;=\;1,\;H_{1}:r\;=\;2$			$H_{0}:r\;=\;2,\;H_{1}:r\;=\;3$		
	LM	$LM_{F}$	LRT	LM	$LM_{F}$	LRT
ER	29.096 *** (0.004)	2.225 *** (0.009)	29.221 *** (0.004)	9.326 (0.675)	0.707 (0.746)	9.338 (0.674)
MAR	17.572 (0.063)	1.606 (0.098)	17.617 (0.062)	-	-	-

Notes: *p* values are in parentheses, *** indicates significance at the 1% level.

**Table 4:** Model parameters determination.

Independent Variable	ER		MAR	
$\left(r,m\right)$	$\left(1,1\right)$	$\left(2,2\right)$	$\left(1,1\right)$	$\left(1,2\right)$
*AIC*	−3.995	−3.996	−4.042	−4.044
*BIC*	−3.970	−3.953	−4.020	−4.007
Whether the location parameter is within the range	No	Yes	No	Yes
$\mathrm{Finally},\;\mathrm{selected}\;\left(r,m\right)$	$\left(2,2\right)$		$\left(1,2\right)$	

**Table 5:** Empirical results of ER based on the PSTR model.

Model	PSTR Model			Fixed Effect Model
	GTFP			GTFP
	(1)			(2)
	β	$\beta^{\left(1\right)}$	$\beta^{\left(2\right)}$	β
ER	0.408 ** (2.28)	−0.173 * (−1.87)	0.122 * (1.80)	0.166 *** (10.02)
urb	−1.114 *** (−5.08)	1.023 *** (4.59)	0.041 (0.93)	0.038 (0.97)
ope	0.610 (0.95)	−0.679 (−1.04)	0.111 (1.08)	−0.062 * (−1.92)
den	−0.818 *** (−2.78)	0.940 *** (3.13)	0.092 * (1.95)	0.786 *** (3.00)
tra	−0.351 (−0.97)	0.476 (1.29)	−0.251 *** (−3.63)	−0.043 * (−1.72)
pos	1.448 *** (3.32)	−0.938 ** (−2.12)	0.164 * (1.79)	0.483 *** (23.86)
$\left(r,m\right)$	$\left(2,2\right)$			-
γ	129.2577			-
c	=0.325, =0.416			-
RSS or R2	61.317			0.3155

Notes: t values are in brackets; ***, **, *, respectively, indicate being significant at the level of 1%, 5%, and 10%.

**Table 6:** Empirical results of MAR based on the PSTR model.

Model	PSTR Model		Fixed Effect Model
	GTFP		GTFP
	(1)		(2)
	β	$\beta^{\left(1\right)}$	β
MAR	1.332 *** (4.41)	0.152 * (1.88)	0.173 *** (8.24)
urb	0.093 (1.01)	0.030 (0.52)	0.024 (0.60)
ope	0.479 *** (2.69)	−0.371 *** (−3.04)	−0.038 (−1.18)
den	−0.354 *** (−2.73)	0.441 *** (3.55)	0.735 *** (2.79)
tra	0.536 *** (3.94)	−0.031 (−0.32)	−0.036 (−1.40)
pos	−0.055 (−0.33)	0.183 * (1.76)	0.478 *** (22.70)
$\left(r,m\right)$	$\left(1,2\right)$		-
γ	47.7281		-
c	c= 0.5092		-
RSS or R2	58.596		0.3085

Notes: t values are in brackets; ***, *, respectively, indicate being significant at the level of 1%, and 10%.

**Table 7:** The test results of the industrial-upgrading effect in policy guidance on GTFP.

Dependent Variable	GTFP			ind	GTFP	
	β	$\beta^{\left(1\right)}$	$\beta^{\left(2\right)}$	β	β	$\beta^{\left(1\right)}$
ER	0.408 ** (2.28)	−0.173 * (−1.87)	0.122 * (1.80)	-	0.018 (0.58)	0.147 (1.53)
ind	-	-	-	0.065 *** (9.29)	0.103 *** (4.19)	0.492 *** (4.43)
urb	−1.114 *** (−5.08)	1.023 *** (4.59)	0.041 (0.93)	0.023 (1.43)	−0.105 *** (−6.31)	−0.357 *** (−5.30)
ope	0.610 (0.95)	−0.679 (−1.04)	0.111 (1.08)	0.086 *** (6.38)	−0.073 *** (−2.60)	0.114 (0.81)
den	−0.818 *** (−2.78)	0.940 *** (3.13)	0.092 * (1.95)	0.814 *** (7.40)	0.107 *** (5.00)	−0.322 *** (−4.39)
tra	−0.351 (−0.97)	0.476 (1.29)	−0.251 *** (−3.63)	0.052 *** (4.88)	0.071 *** (2.62)	−0.222 ** (−2.19)
pos	1.448 *** (3.32)	−0.938 ** (−2.12)	0.164 * (1.79)	0.160 *** (18.83)	0.491 *** (13.32)	0.266 * (1.91)
$\left(r,m\right)$	$\left(2,2\right)$			-	$\left(1,2\right)$	
γ	129.2577			-	46.5565	
c	=0.325, =0.416			-	C = 0.5106	
RSS or R2	61.317			0.2316	60.579	

Notes: t values are in brackets; ***, **, *, respectively, indicate being significant at the level of 1%, 5%, and 10%.

**Table 8:** The test results of the innovation development effect in market driving GTFP.

Dependent Variable	GTFP		inn	GTFP	
	β	$\beta^{\left(1\right)}$	β	β	$\beta^{\left(1\right)}$
MAR	1.332 *** (4.41)	0.152 * (1.88)	-	0.650 ** (2.01)	0.067 (1.61)
inn	-	-	0.063 *** (9.02)	0.210 *** (3.67)	0.346 *** (4.58)
urb	0.093 (1.01)	0.030 (0.52)	0.043 *** (2.63)	0.114 * (1.71)	0.076 (1.53)
ope	0.479 *** (2.69)	−0.371 *** (−3.04)	0.087 *** (6.33)	0.378 ** (2.19)	−0.532 * (−1.74)
den	−0.354 *** (−2.73)	0.441 *** (3.55)	0.633 *** (4.44)	−0.343 *** (−2.67)	0.135 ** (2.55)
tra	0.536 *** (3.94)	−0.031 (−0.32)	0.048 *** (4.48)	0.123 *** (2.87)	0.022 (0.42)
pos	−0.055 (−0.33)	0.183 * (1.76)	0.172 *** (20.44)	0.078 (0.95)	0.142 * (1.13)
$\left(r,m\right)$	$\left(1,2\right)$		-	$\left(1,2\right)$	
γ	47.7281		-	1.1580	
c	c= 0.5092		-	C= 0.4030	
RSS or R2	58.596		0.3718	58.008	

Notes: t values are in brackets; ***, **, *, respectively, indicate being significant at the level of 1%, 5%, and 10%.

## Data Availability

The data presented in this study are available on request from the author.
